# Bis(4-amino-1-hexylpyridinium) bis(1,2-dicyanoethene-1,2-dithiolato)cuprate(II)

**DOI:** 10.1107/S1600536811021611

**Published:** 2011-06-11

**Authors:** Qi Liu, Jianlan Liu

**Affiliations:** a Department of Applied Chemistry, College of Science, Nanjing University of Technology, No.5 Xinmofan Road, Nanjing, Nanjing 210009, People’s Republic of China

## Abstract

The complete complex anion in the title salt, (C_11_H_19_N_2_)_2_[Cu(C_4_N_2_S_2_)_2_], has 2/*m* symmetry while the complete cation is generated by mirror symmetry with the non-H atoms of the alkyl chain lying on the plane. A square-planar geometry based on an S_4_ donor set is found in the anion; the Cu—S distance is 2.2663 (5) Å. In the crystal, inter­molecular N—H⋯N hydrogen bonds link the ions into layers in the *bc* plane comprising alternating rows of cations and anions.

## Related literature

For square-planar *M*[dithiol­ene]_2_ complexes acting as magnetic materials or showing nonlinear optical properties, see: Cassoux *et al.* (1991 ▶); Robertson & Cronin (2002 ▶).
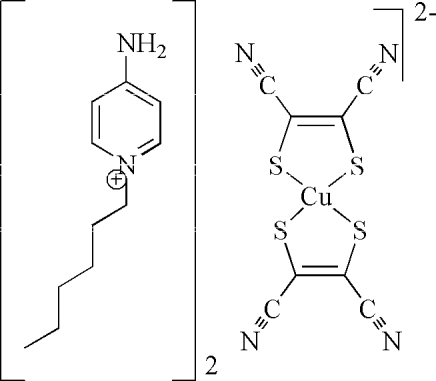


## Experimental

### 

#### Crystal data

(C_11_H_19_N_2_)_2_[Cu(C_4_N_2_S_2_)_2_]
*M* *_r_* = 702.51
Monoclinic, 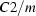

*a* = 13.3648 (9) Å
*b* = 10.0768 (4) Å
*c* = 13.8550 (8) Å
β = 111.902 (8)°
*V* = 1731.24 (17) Å^3^
*Z* = 2
Mo *K*α radiation
μ = 0.91 mm^−1^
*T* = 293 K
0.3 × 0.2 × 0.1 mm

#### Data collection

Siemens SMART CCD area-detector diffractometer
Absorption correction: multi-scan (*SADABS*; Sheldrick, 1996 ▶) *T*_min_ = 0.939, *T*_max_ = 1.000
4194 measured reflections
1805 independent reflections
1299 reflections with *I* > 2σ(*I*)
*R*_int_ = 0.019

#### Refinement

*R*[*F* ^2^ > 2σ(*F* ^2^)] = 0.031
*wR*(*F* ^2^) = 0.074
*S* = 0.92
1805 reflections
117 parameters
H atoms treated by a mixture of independent and constrained refinement
Δρ_max_ = 0.22 e Å^−3^
Δρ_min_ = −0.17 e Å^−3^

### 

Data collection: *SMART* (Siemens, 1996 ▶); cell refinement: *SAINT* (Siemens, 1996 ▶); data reduction: *SAINT*; program(s) used to solve structure: *SHELXS97* (Sheldrick, 2008 ▶); program(s) used to refine structure: *SHELXL97* (Sheldrick, 2008 ▶); molecular graphics: *SHELXTL* (Sheldrick, 2008 ▶); software used to prepare material for publication: *SHELXTL*.

## Supplementary Material

Crystal structure: contains datablock(s) global, I. DOI: 10.1107/S1600536811021611/tk2752sup1.cif

Structure factors: contains datablock(s) I. DOI: 10.1107/S1600536811021611/tk2752Isup2.hkl

Additional supplementary materials:  crystallographic information; 3D view; checkCIF report

## Figures and Tables

**Table 1:** Hydrogen-bond geometry (Å, °)

*D*—H⋯*A*	*D*—H	H⋯*A*	*D*⋯*A*	*D*—H⋯*A*
N2—H2*A*⋯N1^i^	0.81 (2)	2.39 (2)	3.157 (2)	160 (2)

Symmetry code: (i) 
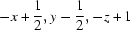
.

**Fig. 1.**
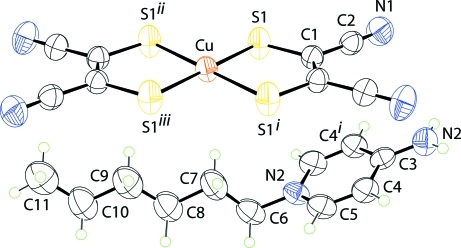
Molecular structure of the ions comprising (I) showing atom labelling. Displacement ellipsoids are drawn at the 50% probability level. The cation has mirror symmetry with i: x, 1-y, z. The Cu atom in the anion is located on a site of symmetry 2/m. Symmetry operations ii: -x, y, -z and iii: -x, 1-y, -z.

**Fig. 2.**
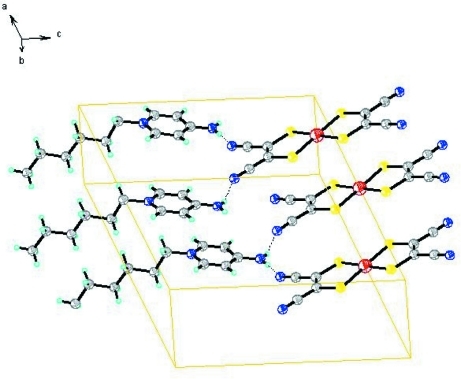
Partial packing view showing the layer in the bc plane. Dashed lines indicate intermolecular N—H···N hydrogen bonds.

## supplementary crystallographic information

### Comment

Square-planar *M*[dithiolene]_2_ complexes have attracted extensive interest in the areas of conducting and magnetic materials, dyes, non-linear optics and catalysis (Robertson *et al.*, 2002; Cassoux *et al.*, 1991). Herein, we report the crystal structure of the title compound, Fig.1.

The [Cu(mnt)_2_]^2-^ dianion is located about a site of symmetry 2/*m*. The 1-hexyl-4-aminopyridinium cation lies on a mirror plane whereby the non-H atoms of the alkyl chain lie on the plane which bisects the 1,4 atoms of the benzene ring.

In the crystal structure, intermolecular N—H···N hydrogen bonds (Table 1) link the cations and anions to form a layer in the *bc* plane comprising alternating cations and anions.

### Experimental

Disodium maleonitriledithiolate (468 mg, 2.5 mmol) and cupric nitrate trihydrate (302 mg, 1.25 mmol) were mixed under stirring in water (20 mL) at room temperature. Subsequently, a solution of 1-hexyl-4-aminopyridinium iodide (765 mg, 2.5 mmol) in water (10 mL) was added to the mixture. The brown precipitate that formed immediately was filtered off and washed with water. The crude product was recrystallized from acetone (20 mL) to give brown crystals. The crystals suitable for X-ray diffraction measurements were obtained by diffusing diethyl ether into the solution of the salt in acetone for 6 days.

### Refinement

The C-bound H atoms were geometrically placed (C—H = 0.93 or 0.96 Å) and refined as riding with *U_iso_*(H) = 1.2-1.5*U_eq_*(C). The N-bound H atom was refined freely.

### Crystal data

**Table:**

(C_11_H_19_N_2_)_2_[Cu(C_4_N_2_S_2_)_2_]	*F*(000) = 734
*M**_r_* = 702.51	*D*_x_ = 1.348 Mg m^−^^3^
Monoclinic, *C*2/*m*	Melting point = 430–432 K
Hall symbol: -C 2y	Mo *K*α radiation, λ = 0.71073 Å
*a* = 13.3648 (9) Å	Cell parameters from 2059 reflections
*b* = 10.0768 (4) Å	θ = 3.1–29.2°
*c* = 13.8550 (8) Å	µ = 0.91 mm^−^^1^
β = 111.902 (8)°	*T* = 293 K
*V* = 1731.24 (17) Å^3^	Block, brown
*Z* = 2	0.3 × 0.2 × 0.1 mm

### Data collection

**Table:**

Siemens SMART CCD area-detector diffractometer	1805 independent reflections
Radiation source: fine-focus sealed tube	1299 reflections with *I* > 2σ(*I*)
graphite	*R*_int_ = 0.019
φ and ω scans	θ_max_ = 26.0°, θ_min_ = 3.1°
Absorption correction: multi-scan (*SADABS*; Sheldrick, 1996)	*h* = −12→16
*T*_min_ = 0.939, *T*_max_ = 1.000	*k* = −11→12
4194 measured reflections	*l* = −17→16

### Refinement

**Table:**

Refinement on *F*^2^	Primary atom site location: structure-invariant direct methods
Least-squares matrix: full	Secondary atom site location: difference Fourier map
*R*[*F*^2^ > 2σ(*F*^2^)] = 0.031	Hydrogen site location: inferred from neighbouring sites
*wR*(*F*^2^) = 0.074	H atoms treated by a mixture of independent and constrained refinement
*S* = 0.92	*w* = 1/[σ^2^(*F*_o_^2^) + (0.0411*P*)^2^] where *P* = (*F*_o_^2^ + 2*F*_c_^2^)/3
1805 reflections	(Δ/σ)_max_ < 0.001
117 parameters	Δρ_max_ = 0.22 e Å^−^^3^
0 restraints	Δρ_min_ = −0.17 e Å^−^^3^

### Special details

**Table:**

Geometry. All e.s.d.'s (except the e.s.d. in the dihedral angle between two l.s. planes) are estimated using the full covariance matrix. The cell e.s.d.'s are taken into account individually in the estimation of e.s.d.'s in distances, angles and torsion angles; correlations between e.s.d.'s in cell parameters are only used when they are defined by crystal symmetry. An approximate (isotropic) treatment of cell e.s.d.'s is used for estimating e.s.d.'s involving l.s. planes.
Refinement. Refinement of *F*^2^ against ALL reflections. The weighted *R*-factor *wR* and goodness of fit *S* are based on *F*^2^, conventional *R*-factors *R* are based on *F*, with *F* set to zero for negative *F*^2^. The threshold expression of *F*^2^ > σ(*F*^2^) is used only for calculating *R*-factors(gt) *etc*. and is not relevant to the choice of reflections for refinement. *R*-factors based on *F*^2^ are statistically about twice as large as those based on *F*, and *R*- factors based on ALL data will be even larger.

### Fractional atomic coordinates and isotropic or equivalent isotropic displacement parameters (Å^2^)

**Table:**

	*x*	*y*	*z*	*U*_iso_*/*U*_eq_	
Cu1	0.0000	0.5000	0.0000	0.04510 (17)	
S1	0.04362 (5)	0.66007 (5)	0.12376 (4)	0.0623 (2)	
N1	0.12555 (15)	0.7075 (2)	0.40284 (14)	0.0721 (6)	
N2	0.3513 (2)	0.5000	0.5099 (2)	0.0615 (8)	
N3	0.39247 (19)	0.5000	0.23171 (18)	0.0536 (6)	
C1	0.07750 (14)	0.56692 (18)	0.23653 (14)	0.0445 (4)	
C2	0.10516 (16)	0.6424 (2)	0.33068 (15)	0.0507 (5)	
C3	0.3637 (2)	0.5000	0.4193 (2)	0.0472 (7)	
C4	0.37096 (16)	0.38155 (19)	0.36957 (15)	0.0544 (5)	
H4A	0.3666	0.3005	0.3998	0.065*	
C5	0.38435 (18)	0.3850 (2)	0.27783 (17)	0.0586 (6)	
H5A	0.3881	0.3054	0.2454	0.070*	
C6	0.4079 (3)	0.5000	0.1312 (2)	0.0678 (9)	
H6A	0.4487	0.4229	0.1280	0.081*	
C7	0.3047 (3)	0.5000	0.0398 (3)	0.0810 (10)	
H7A	0.2635	0.4226	0.0414	0.097*	
C8	0.3282 (3)	0.5000	−0.0612 (3)	0.0819 (11)	
H8A	0.3740	0.4117	−0.0657	0.098*	
C9	0.2314 (3)	0.5000	−0.1571 (3)	0.0917 (12)	
H9A	0.1892	0.4230	−0.1569	0.110*	
C10	0.2529 (4)	0.5000	−0.2572 (3)	0.0930 (12)	
H10A	0.2950	0.4229	−0.2577	0.112*	
C11	0.1534 (3)	0.5000	−0.3517 (3)	0.0931 (12)	
H11A	0.1716	0.5000	−0.4125	0.140*	
H11B	0.1119	0.4222	−0.3519	0.140*	
H2A	0.3461 (18)	0.432 (2)	0.5381 (16)	0.075 (8)*	

### Atomic displacement parameters (Å^2^)

**Table:**

	*U*^11^	*U*^22^	*U*^33^	*U*^12^	*U*^13^	*U*^23^
Cu1	0.0613 (3)	0.0335 (3)	0.0420 (3)	0.000	0.0211 (2)	0.000
S1	0.1042 (5)	0.0323 (3)	0.0463 (3)	0.0005 (3)	0.0235 (3)	0.0001 (2)
N1	0.0822 (14)	0.0679 (13)	0.0574 (11)	0.0058 (11)	0.0159 (10)	−0.0184 (10)
N2	0.076 (2)	0.0505 (19)	0.0559 (16)	0.000	0.0218 (15)	0.000
N3	0.0594 (15)	0.0435 (14)	0.0669 (15)	0.000	0.0340 (13)	0.000
C1	0.0492 (11)	0.0407 (10)	0.0423 (10)	0.0004 (9)	0.0155 (9)	−0.0020 (8)
C2	0.0538 (13)	0.0454 (11)	0.0486 (11)	0.0054 (10)	0.0143 (10)	0.0004 (10)
C3	0.0386 (16)	0.0423 (17)	0.0544 (17)	0.000	0.0100 (14)	0.000
C4	0.0649 (14)	0.0343 (11)	0.0652 (14)	0.0001 (10)	0.0257 (12)	0.0042 (10)
C5	0.0692 (15)	0.0352 (11)	0.0769 (15)	0.0012 (10)	0.0338 (13)	−0.0054 (11)
C6	0.080 (2)	0.058 (2)	0.083 (2)	0.000	0.051 (2)	0.000
C7	0.090 (3)	0.096 (3)	0.070 (2)	0.000	0.045 (2)	0.000
C8	0.092 (3)	0.087 (3)	0.079 (2)	0.000	0.046 (2)	0.000
C9	0.108 (3)	0.096 (3)	0.084 (3)	0.000	0.051 (3)	0.000
C10	0.114 (3)	0.099 (3)	0.080 (3)	0.000	0.053 (3)	0.000
C11	0.122 (3)	0.073 (3)	0.097 (3)	0.000	0.055 (3)	0.000

### Geometric parameters (Å, °)

**Table:**

Cu1—S1^i^	2.2663 (5)	C4—C5	1.349 (3)
Cu1—S1	2.2663 (5)	C4—H4A	0.9300
Cu1—S1^ii^	2.2663 (5)	C5—H5A	0.9300
Cu1—S1^iii^	2.2663 (5)	C6—C7	1.483 (4)
S1—C1	1.7319 (19)	C6—H6A	0.9600
N1—C2	1.141 (2)	C7—C8	1.545 (4)
N2—C3	1.327 (4)	C7—H7A	0.9600
N2—H2A	0.80 (2)	C8—C9	1.468 (5)
N3—C5	1.347 (2)	C8—H8A	1.0943
N3—C5^iii^	1.347 (2)	C9—C10	1.519 (4)
N3—C6	1.482 (3)	C9—H9A	0.9601
C1—C1^iii^	1.349 (4)	C10—C11	1.478 (5)
C1—C2	1.434 (3)	C10—H10A	0.9600
C3—C4	1.399 (2)	C11—H11A	0.9600
C3—C4^iii^	1.399 (2)	C11—H11B	0.9600
			
S1^i^—Cu1—S1	180.00 (2)	N3—C5—C4	122.1 (2)
S1^i^—Cu1—S1^ii^	90.75 (3)	N3—C5—H5A	118.9
S1—Cu1—S1^ii^	89.25 (3)	C4—C5—H5A	118.9
S1^i^—Cu1—S1^iii^	89.25 (3)	N3—C6—C7	113.0 (2)
S1—Cu1—S1^iii^	90.75 (3)	N3—C6—H6A	108.9
S1^ii^—Cu1—S1^iii^	180.00 (3)	C7—C6—H6A	108.9
C1—S1—Cu1	101.76 (6)	C6—C7—C8	109.6 (3)
C3—N2—H2A	121.7 (17)	C6—C7—H7A	109.8
C5—N3—C5^iii^	118.7 (2)	C8—C7—H7A	109.5
C5—N3—C6	120.65 (12)	C9—C8—C7	114.3 (3)
C5^iii^—N3—C6	120.65 (12)	C9—C8—H8A	105.7
C1^iii^—C1—C2	122.02 (11)	C7—C8—H8A	111.0
C1^iii^—C1—S1	122.82 (6)	C8—C9—C10	115.0 (3)
C2—C1—S1	115.16 (14)	C8—C9—H9A	108.5
N1—C2—C1	176.9 (2)	C10—C9—H9A	108.3
N2—C3—C4	121.46 (13)	C11—C10—C9	113.2 (3)
N2—C3—C4^iii^	121.46 (13)	C11—C10—H10A	109.3
C4—C3—C4^iii^	117.1 (3)	C9—C10—H10A	108.5
C5—C4—C3	120.0 (2)	C10—C11—H11A	109.8
C5—C4—H4A	120.0	C10—C11—H11B	109.3
C3—C4—H4A	120.0	H11A—C11—H11B	109.5

Symmetry codes: (i) −*x*, −*y*+1, −*z*; (ii) −*x*, *y*, −*z*; (iii) *x*, −*y*+1, *z*.

### Hydrogen-bond geometry (Å, °)

**Table:**

*D*—H···*A*	*D*—H	H···*A*	*D*···*A*	*D*—H···*A*
N2—H2A···N1^iv^	0.81 (2)	2.39 (2)	3.157 (2)	160 (2)

Symmetry codes: (iv) −*x*+1/2, *y*−1/2, −*z*+1.
